# A case report of Huntington’s disease presenting with psychiatric symptoms as initial manifestation combined with memory decline and motor dysfunction

**DOI:** 10.1007/s10072-026-08987-5

**Published:** 2026-04-18

**Authors:** Aifan Li, Qiong Zhang, Yongfang Li, Dongzhi Yang

**Affiliations:** 1 Cognitive Dysfunction Ward of Neurology Department, Zhengzhou First People’s Hospital, Zhengzhou, 450000 China; 2 Department of Medical Genetics & Cell Biology, School of Basic Medical Sciences, Zhengzhou University, Zhengzhou, 450001 China; 3 School of Life Sciences, Zhengzhou University, Zhengzhou, 450001 China

**Keywords:** Huntington’s disease, Genetic testing, Inheritance, Psychiatric symptoms, Cognitive impairment

To the Editor,

Huntington’s disease (HD) is an autosomal dominant neurodegenerative disorder classically characterized by a triad of chorea, cognitive decline, and psychiatric disturbances. While motor symptoms often prompt initial clinical suspicion, psychiatric and cognitive manifestations can precede them by years, frequently leading to diagnostic delays and mismanagement [1]. We report a genetically confirmed case of HD where psychiatric symptoms were the initial and predominant feature for nearly a decade, underscoring the critical importance of early genetic testing in atypical presentations.

A 54-year-old man presented to our clinic in August 2024 with a complex 14-year history. His illness began at age 40 with progressive personality changes, marked by increasing irritability, social withdrawal, and emotional lability. Due to the insidious nature of these early symptoms, he did not seek medical attention at that time, and no formal psychiatric evaluation was conducted. Approximately nine years ago (at age 45), he and his family noted a progressive decline in memory and executive function, impacting his daily work. Involuntary movements first appeared five years ago (at age 49), gradually worsening and involving his limbs and face. His family history was significant for unspecified psychiatric illness and premature death (at age 50) in his mother, suggesting possible undiagnosed neurological disease.

**Fig. 1:**
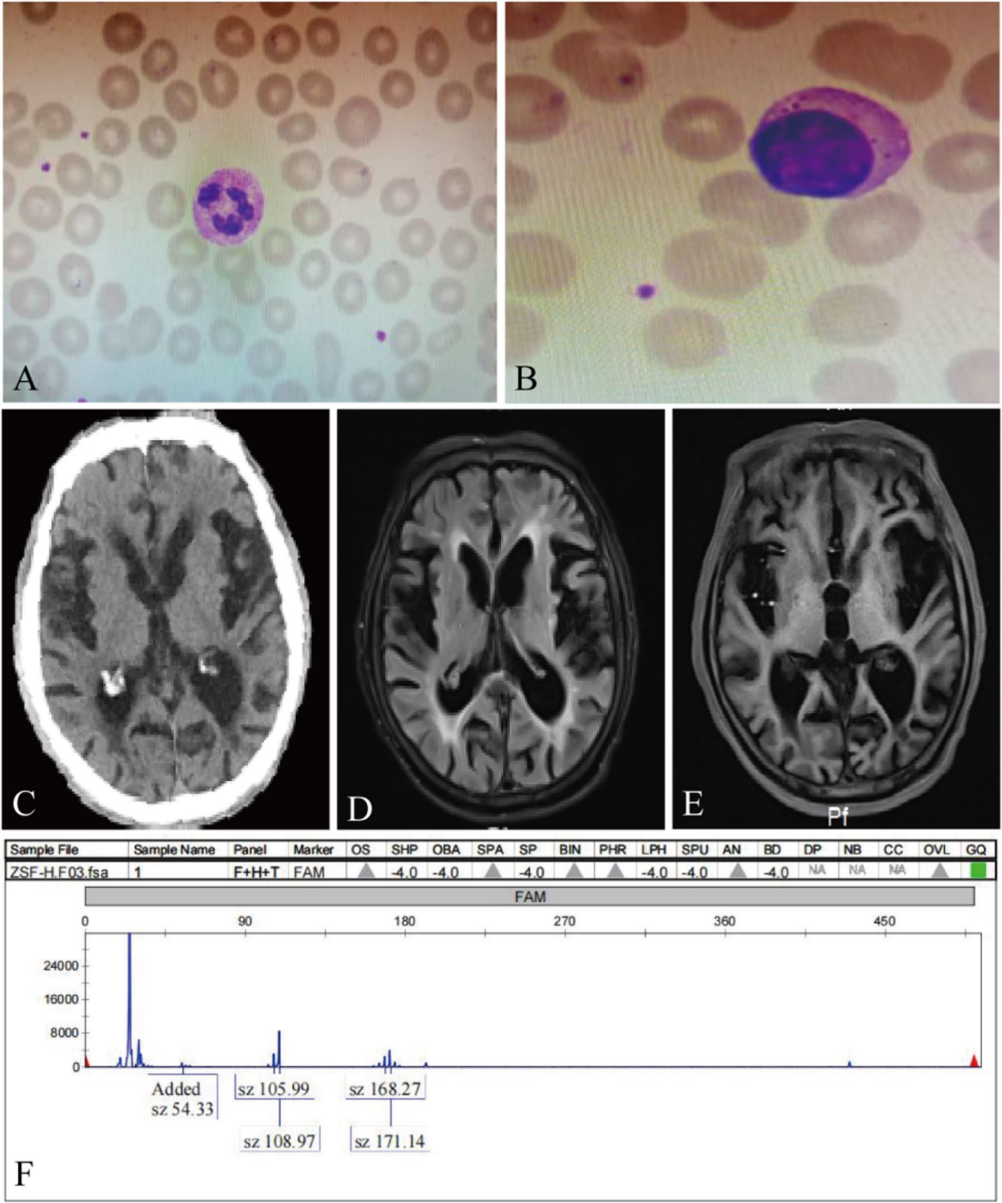
Neuroimaging, peripheral blood smear, and genetic findings in a patient with Huntington’s disease. (**A**-**B**) Peripheral blood smear (Wright-Giemsa stain) showing normocytic erythrocytes without acanthocytes. (**C**) Non-contrast brain CT scan demonstrating generalized cerebral atrophy with prominent sulci and ventricular enlargement. (**D**) FLAIR image showing diffuse cerebral atrophy and white matter hyperintensities. (**E**) T1-weighted image confirming volume loss in the caudate nuclei. (**F**) Electropherogram of the HTT gene analysis: pathogenic expansion of CAG repeats (*n* = 46) in one allele and normal repeats (*n* = 25) in the other

Neurological examination on admission revealed generalized choreoathetoid movements, dysarthria, and significant cognitive impairment that precluded formal MMSE testing. Bilateral pathological reflexes were absent. A peripheral blood smear showed normocytic erythrocytes without acanthocytes (Fig. 1A-B), effectively ruling out neuroacanthocytosis, a key differential diagnosis for HD. Brain magnetic resonance imaging (MRI) demonstrated diffuse cerebral atrophy, marked bilateral atrophy of the caudate nucleus with consequent enlargement of the frontal horns of the lateral ventricles (giving a “box-like” appearance), and non-specific periventricular white matter hyperintensities (Fig. 1C-E). Genetic testing, prompted by the clinical and radiological findings, confirmed the diagnosis of HD. Analysis of the *HTT* gene revealed a pathogenic allele with 46 CAG trinucleotide repeats (full penetrance, as normal is < 36) and a normal allele with 25 repeats (Fig. 1F).

Symptomatic treatment was initiated with haloperidol (2 mg/day) for chorea and a combination of olanzapine (5 mg/day) and sertraline (50 mg/day) for depression and irritability. While the choreiform movements showed moderate improvement, the patient’s depressive symptoms and apathy proved largely refractory to pharmacological intervention.

This case provides a striking illustration of the diagnostic odyssey often associated with Huntington’s disease. The patient’s initial psychiatric symptoms (personality changes) predated the onset of motor dysfunction by a full nine years, a latency period consistent with studies reporting that up to 42.4% of HD patients exhibit neuropsychiatric symptoms prior to the development of chorea [2]. This prolonged “psychiatric prodrome” is a major contributor to diagnostic delay. In this instance, the delay was compounded by the patient’s initial failure to seek care and the absence of early specialist evaluation, leading to over a decade of uncertainty before a definitive diagnosis was made.

The case also highlights the critical role of genetic testing. While MRI findings of caudate atrophy are highly characteristic of HD, their absence in early stages is well-documented. Relying solely on imaging in patients presenting with unexplained psychiatric or cognitive decline would miss the diagnosis. This reinforces the necessity of maintaining a high index of suspicion for HD in at-risk individuals, particularly those with a suggestive family history, and proceeding directly to genetic testing [3].

Currently, HD management remains entirely symptomatic. Although antipsychotics like haloperidol can be effective in managing chorea, psychiatric symptoms often show a variable and incomplete response to standard treatments [4]. The poor response of depressive symptoms in our patient aligns with recent findings suggesting that psychiatric symptoms in HD, such as depression and irritability, may follow distinct neurobiological trajectories [5]. This indicates that the psychopathological mechanisms in HD likely differ from those in primary psychiatric disorders. It explains why standard treatment strategies may have limited efficacy and underscores the urgent, unmet need for targeted therapies based on the underlying pathophysiology of HD, rather than simply its phenomenological presentation.

Huntington’s disease should be considered in the differential diagnosis for any adult presenting with progressive psychiatric symptoms and cognitive decline, especially when there is a family history of neurological or psychiatric disorders. While no single psychiatric phenotype is pathognomonic for HD, a constellation of features should raise suspicion. These include the insidious emergence of neuropsychiatric symptoms such as emotional lability, irritability, apathy, and executive dysfunction without significant psychosocial precipitants; a poor response to conventional psychiatric treatments (e.g., antidepressants alone); and a slowly progressive course [6]. For such patients, genetic testing for HD is not merely an option, but an imperative. A definitive diagnosis is essential not only for guiding symptomatic management and enabling informed family planning and genetic counseling, but also for ending the often-protracted diagnostic uncertainty for the patient and their family.
